# A210 BARRIERS TO ACCESSING INTERVENTIONS FOR PSYCHOLOGICAL DISTRESS IN PERSONS LIVING WITH IBD

**DOI:** 10.1093/jcag/gwae059.210

**Published:** 2025-02-10

**Authors:** N Willett, C Heisler, N Rohatinsky, S Farina, M Stewart, M Vallis, T Shepherd, T Huard, E Neil, J Jones

**Affiliations:** Division of Digestive Care and Endoscopy, Nova Scotia Health Authority, Halifax, NS, Canada; Division of Digestive Care and Endoscopy, Nova Scotia Health Authority, Halifax, NS, Canada; University of Saskatchewan, Saskatoon, SK, Canada; Division of Digestive Care and Endoscopy, Nova Scotia Health Authority, Halifax, NS, Canada; Division of Digestive Care and Endoscopy, Nova Scotia Health Authority, Halifax, NS, Canada; Dalhousie University, Halifax, NS, Canada; Division of Digestive Care and Endoscopy, Nova Scotia Health Authority, Halifax, NS, Canada; Division of Digestive Care and Endoscopy, Nova Scotia Health Authority, Halifax, NS, Canada; Division of Digestive Care and Endoscopy, Nova Scotia Health Authority, Halifax, NS, Canada; Division of Digestive Care and Endoscopy, Nova Scotia Health Authority, Halifax, NS, Canada

## Abstract

**Background:**

Inflammatory Bowel Disease-related psychological distress (IBD-PD) refers to the emotional impact of IBD and is associated with mental health disorders, increased IBD activity, and premature mortality. Up to 90% of IBD patients experience some form of IBD-PD. The inability to provide person-centered care for IBD-PD that is proportional to clinical need is a critical issue.

**Aims:**

The aim of this research initiative was to identify barriers to accessing evidence-based interventions for mental health support for IBD-PD.

**Methods:**

This was a qualitative research study in which virtual semi-structured interviews took place between Oct 2021 and Mar 2022. The semi-structured interview script was developed with researchers, IBD care providers, and patient research partners and was guided by the COM-B Behaviour Change Wheel framework and the Theoretical Domains Framework (TDF). Adults (> 18 years of age) with a confirmed diagnosis of IBD were recruited from IBD clinics. Using thematic analysis as per Braun and Clarke (2006), codes were generated to identify themes.

**Results:**

The total number of participants recruited was 14. The mean participant age was 37.6 years (range 23-57) with 57.1% as female (8/14). In terms of geographical locale, 28.6% lived rurally (4/14) and 71.4% (10/14) lived in an urban area. Thematic analyses identified the following themes: 1) Mental health is an important part of integrated IBD care. 2) Barriers to accessing psychological supports persist and include stigma, ignorance of IBD and mental health, costs and insurance coverage, and limited access and availability; 3) Health care providers should do more to address and refer patients to mental health supports.

**Conclusions:**

From the perspective of the patient, accessing care for IBD-PD is challenging due to financial, physical, administrative, sociocultural, and informational barriers. Patients’ previous interactions with the healthcare system and mental health care providers color their view on mental health care and influence their preferences for context, setting and delivery mode for interventions. It is important to engage patients in the design and implementation of health service interventions. The information they share highlights barriers patients frequently encounter, and which aspects of care can be improved to meet patient-specific needs and match patient preferences.

**Funding Agencies:**

None

